# Whole Genome Linkage Disequilibrium and Effective Population Size in a Coho Salmon (*Oncorhynchus kisutch*) Breeding Population Using a High-Density SNP Array

**DOI:** 10.3389/fgene.2019.00498

**Published:** 2019-05-22

**Authors:** Agustín Barría, Kris A. Christensen, Grazyella Yoshida, Ana Jedlicki, Jong S. Leong, Eric B. Rondeau, Jean P. Lhorente, Ben F. Koop, William S. Davidson, José M. Yáñez

**Affiliations:** ^1^ Facultad de Ciencias Veterinarias y Pecuarias, Universidad de Chile, Santiago, Chile; ^2^ Department of Biology, Centre for Biomedical Research, University of Victoria, Victoria, BC, Canada; ^3^ Benchmark Genetics Chile, Puerto Montt, Chile; ^4^ Department of Molecular Biology and Biochemistry, Simon Fraser University, Burnaby, BC, Canada; ^5^ Nucleo Milenio INVASAL, Concepcion, Chile

**Keywords:** linkage disequilibrium, *Oncorhynchus kisutch*, selective breeding, GWAS, effective population size

## Abstract

The estimation of linkage disequilibrium between molecular markers within a population is critical when establishing the minimum number of markers required for association studies, genomic selection, and inferring historical events influencing different populations. This work aimed to evaluate the extent and decay of linkage disequilibrium in a coho salmon breeding population using a high-density SNP array. Linkage disequilibrium was estimated between a total of 93,502 SNPs found in 64 individuals (33 dams and 31 sires) from the breeding population. The markers encompass all 30 coho salmon chromosomes and comprise 1,684.62 Mb of the genome. The average density of markers per chromosome ranged from 48.31 to 66 per 1 Mb. The minor allele frequency averaged 0.26 (with a range from 0.22 to 0.27). The overall average linkage disequilibrium among SNPs pairs measured as *r*^2^ was 0.10. The Average *r*^2^ value decreased with increasing physical distance, with values ranging from 0.21 to 0.07 at a distance lower than 1 kb and up to 10 Mb, respectively. An *r*^2^ threshold of 0.2 was reached at distance of approximately 40 Kb. Chromosomes Okis05, Okis15 and Okis28 showed high levels of linkage disequilibrium (>0.20 at distances lower than 1 Mb). Average *r*^2^ values were lower than 0.15 for all chromosomes at distances greater than 4 Mb. An effective population size of 43 was estimated for the population 10 generations ago, and 325, for 139 generations ago. Based on the effective number of chromosome segments, we suggest that at least 74,000 SNPs would be necessary for an association mapping study and genomic predictions. Therefore, the SNP panel used allowed us to capture high-resolution information in the farmed coho salmon population. Furthermore, based on the contemporary *N*_e_, a new mate allocation strategy is suggested to increase the effective population size.

## Background

Coho salmon (*Oncorhynchus kisutch*) is one of the six Pacific salmon species found in North American and Asian watersheds (Groot and Margolis, 1991). This species was introduced into Chilean streams during the 1920s promoted by the Chilean Institute of Fisheries Department. Cultivation of coho salmon began in Chile at the end of the 1970s, when Chile imported almost 500,000 eggs from the Kitimat river (British Columbia) and Oregon, becoming the genetic basis of the broodstocks in Chile (Neira et al., 2014). Twenty years later, the production of the first eggs for commercial use was produced in Chile (SalmonChile, 2007). Currently, Chile is the main producer of farmed coho salmon, with the production of nearly 160,000 tons in 2014 (FAO, 2016). This represents more than 90% of the global farmed coho production (Canada and Japan are the other major coho salmon producers) (FAO, 2016). The temperature and the quality of the Chilean freshwater environments have reduced the coho reproductive cycle to only 2 years (Estay et al., 1997). To date, numerous genetic programs have been developed for coho salmon in Chile. These programs are mainly focused on growth, disease resistance, and flesh color (Neira et al., 2014).

With the advent of next generation sequencing (NGS) and high-throughput genotyping technologies, it has become possible to perform artificial selection through the use of genomic estimated breeding values (GEBVs). By using dense molecular markers from the whole genome, genomic selection (GS) can be used in broodstock enhancement (Bennewitz et al., 2009). This methodology makes it possible to estimate GEBVs with high accuracy, even with animals without recorded phenotypes (Meuwissen et al., 2001), which has improved the accuracy of selection in salmonid species (Ødegård et al., 2014; Tsai et al., 2016; Bangera et al., 2017; Correa et al., 2017; Yoshida et al., 2018; Barría et al., 2018a). Genome wide association studies (GWAs) and GS, exploit linkage disequilibrium (LD) between molecular markers. The amount of LD between loci is important in GWAs, as the extent of LD indicates the necessary number of SNPs to assure that causative mutations are in LD with genetic markers (Flint-Garcia et al., 2003). GWAs are key for mapping traits with commercial interest to specific variants in the genome. For GS, LD is related to the likelihood of successfully tagging the SNP effect in genomic breeding value prediction (Kemper and Goddard, 2012).

LD allows researchers to explore the genetic basis of traits influencing productivity. Through the comparison of the extent and pattern of LD, it is possible to elucidate the diversity among breeds with different phenotypic attributes, and even identify genomic regions subject to different selective pressures (McKay et al., 2007; López et al., 2015). The most common LD measurements are *r*^2^ and |D′|, both ranging from 0 to 1. When |D′| < 1, it indicates the occurrence of historical recombination between loci, while |D′| = 1 indicates no recombination. The *r*^2^ statistic represents the correlation between genotypes from molecular marker pairs. This latter parameter is preferred over |D′| because |D′| tends to be overestimated in small samples sizes and when low-frequency alleles are used (Teare et al., 2002). Moreover, in association studies, *r*^2^ is preferred due to the inverse relationship between its value and the sample size needed to detect a significant association between a causative variant and molecular markers (Wall and Pritchard, 2003).

Despite the many GWAs and GS analyses performed in Atlantic salmon (Gutierrez et al., 2015; Tsai et al., 2015, 2016; Bangera et al., 2017; Correa et al., 2017), rainbow trout (Vallejo et al., 2016, 2017) and coho salmon (Barría et al., 2018a), none of them have evaluated the LD in the studied populations. Further, most of the linkage disequilibrium studies have been focused on the extent and decay pattern of LD in livestock species, such as dairy (Sargolzaei et al., 2008; Bohmanova et al., 2010) and beef cattle (McKay et al., 2007; Makina et al., 2015), plants (Delourme et al., 2013; Porto-Neto et al., 2014), and pigs (Saura et al., 2015). Recently, LD has been evaluated in farmed rainbow trout (*Oncorhynchus mykiss*) (Rexroad and Vallejo, 2009) and in Atlantic salmon (Kijas et al., 2017; Barría et al., 2018b).

The first step to calculate the number of molecular markers necessary for genomic selection and association mapping is to estimate the extent and decline of LD within a population. To date, there have been no studies aimed to characterize the levels and extent of LD in coho salmon. The current work aimed to evaluate the effective population size and the extent of linkage disequilibrium, at the genomic and chromosome level, on a breeding coho salmon population using a 200K high-density SNP chip array.

## Materials and Methods

### Populations and Samples

The coho salmon samples were obtained from a breeding population belonging to a genetic improvement program established in 1998 run by Pesquera Antares (Puerto Montt, Chile). Using *best linear unbiased prediction* (BLUP), harvest weight had been selected over eight generations in this population. For LD estimations, a total of 64 animals (33 sires and 31 dams), corresponding to the parents of 33 families from a 2012-spawning year class, were selected. The mean relatedness among individuals (0.07) was estimated using Plink v1.09 (Purcell et al., 2007). For specific details about reproductive management, mating design, rearing conditions and inbreeding and breeding objectives of the genetic program for this population see Dufflocq et al. (2016) and Yáñez et al. (2014, 2016). Sampling protocols were approved by the Animal Bioethics Committee from Universidad de Chile (No. 08-2015).

### Genotyping

Genomic DNA was extracted from fin clips from the 64 individuals. Genotyping was carried out using a 200K Affymetrix Axiom® myDesign Custom Array developed for coho salmon by the EPIC4 genome consortium^1^ and constructed by ThermoFisher Scientific. Subsequent work will describe the design and performance of the array in greater detail, but in brief: This dense SNP array contains 203,077 polymorphic SNPs. Genotyping of the SNP array was performed by the McGill University and Genome Quebec Innovation Centre. Genotype calling was performed using Axiom Analysis Suite v3.1 (Thermo Scientific) following the Axiom Analysis user guide. A total of 22 SNPs were subsequently discarded prior to analysis due to unknown position on the coho salmon reference genome (GCF_002021735.1); a further 35,569 markers were discarded as they were identified as problematic (OTV, Call Rate Below Threshold, Other). This left 167,486 SNP markers for further consideration. The following parameters were used to exclude low-confidence SNPs using plink software: Hardy-Weinberg Equilibrium (HWE) *p* < 3.8e−7, Minor Allele Frequency (MAF) ≤ 0.05 and genotyping call rate < 0.95. Fish with genotyping call rates <0.95 were excluded from further analyses. Subsequent analyses were assessed using the SNPs markers which passed all quality control (QC) criteria.

### LD Estimation

The LD between each pair of genetic markers was estimated using Pearson’s squared correlation coefficient (*r*^2^) statistic which is less sensitive to allelic frequencies (Ardlie et al., 2002), more suitable for biallelic markers (Zhao et al., 2005) and allows to compare estimations with previous studies. Pair-wise LD as *r*^2^ values, were estimated with Plink v1.09 (Purcell et al., 2007), based on the formula proposed by Hill and Robertson (1968). Genotypes were coded as 0, 1, and 2 relative to the number of non-reference alleles. The parameter -inter-chr, in conjunction with a ld-window-*r*^2^ set to zero, was used to obtain correlations between all the pairs of SNPs within each chromosome independently of their *r*^2^ value. Based on the physical distance, we created bins of 100 kb for each SNP pair. Decay and extend of the LD was estimated. LD decay curves for SNP pair were calculated as the average *r*^2^ within each bin, up to a distance of 10 Mb. Average *r*^2^ per chromosome was calculated sorting SNPs pairs into 10 bins according to an increasing average distance. The used distance was from 0 to 0.99 Mb and from 9 to 10 Mb (in the first and last bin, respectively), between SNPs pairs on each chromosome.

### Effective Population Size and Number of Chromosome Segments

Contemporary effective population size (*N*_e_) was estimated using NeEstimator v2.01 (Do et al., 2014). Estimation was assessed based on LD method (Waples and Do, 2008), setting a critical value of 0.05 and using a non-random mating model.

The historical effective population size was estimated using SNeP software v1.1 (Barbato et al., 2015). Based on estimated LD values, historical population size estimation was calculated with the following equation proposed by Corbin et al. (2012):

$$N_{t}=\frac{1}{\left(4f\left(c_{t}\right)\right)}\left(\frac{1}{E\left[r_{adj}^{2}|c_{t}\right]}-\alpha \right)$$

where *N_t_* and *c_t_* refers to the effective population size and recombination rate, t generations ago, respectively. Being the latter, proportional to the physical distance between the SNPs, $r_{adj}^{2}$ is the adjusted estimation of LD based on sample size, and *α* refers to the adjustment for mutation rate. Considering that mutation does occurs, we used an *α* = 2 (Tenesa et al., 2007; Vallejo et al., 2018). *N*_e_ estimation was calculated with a minimum and maximum distance between SNPs of 0 and 5 Mb, respectively. Data was arranged in 30 bins of 50 kb distance each. Thus, *N*_e_ was calculated from the *r*^2^ estimated for the average distance of each bin.

Effective number of chromosome segments (*M*_e_) was estimated based on the following formula proposed by Goddard et al. (2011).

$$M_{e}=1/\mathrm{mean}\left(r^{2}\right)$$

where mean(*r*^2^) refers to the average linkage disequilibrium over all pairwise combination of SNPs within each chromosome. Thus, the total *M_e_* within the population is the sum of each individual *M_e_*.

## Results

### SNPs Quality Control

No individuals were removed after QC. From the 167,486 SNPs anchored to chromosomes, a total of 93,502 SNPs passed the quality control and were identified as segregating along the coho salmon genome. The MAF distribution of the identified SNPs was nearly uniform along the 30 chromosomes, with an average of 0.26 ± 0.01 (mean ± standard deviation), and a minimum and maximum value of 0.22 and 0.27, respectively (Table 1).

**Table 1:** Summary statistics for the evaluated SNPs and linkage disequilibrium values along coho salmon chromosomes.

Okis	Length (Mb)	Number of SNPs	SNP density (Mb)	Mean (*r*^2^)	Median (*r*^2^)	SD (*r*^2^)	MAF
01	67.36	3,840	57.01	0.12	0.06	0.16	0.27
02	74.29	3,990	53.71	0.10	0.05	0.13	0.26
03	70.07	3,882	55.40	0.12	0.06	0.10	0.27
04	79.83	4,846	60.70	0.10	0.05	0.14	0.26
05	71.75	3,748	52.24	0.13	0.06	0.17	0.22
06	76.69	3,854	50.25	0.13	0.05	0.18	0.27
07	50.39	2,746	54.49	0.11	0.05	0.15	0.27
08	67.50	3,806	56.39	0.12	0.06	0.16	0.27
09	39.42	2,166	54.95	0.07	0.03	0.11	0.25
10	65.20	3,449	52.90	0.10	0.05	0.14	0.26
11	79.38	4,036	50.84	0.14	0.07	0.18	0.27
12	51.25	3,135	61.17	0.09	0.04	0.12	0.26
13	66.77	3,755	56.24	0.09	0.04	0.13	0.26
14	71.35	3,712	52.03	0.11	0.06	0.14	0.27
15	66.87	3,662	54.76	0.10	0.05	0.13	0.26
16	33.63	1,954	58.10	0.08	0.04	0.11	0.26
17	75.53	4,200	55.61	0.12	0.06	0.16	0.27
18	66.40	3,570	53.77	0.10	0.05	0.13	0.25
19	54.89	2,877	52.41	0.11	0.05	0.15	0.26
20	40.41	2,333	57.73	0.08	0.03	0.12	0.26
21	34.95	1,921	54.96	0.10	0.05	0.14	0.27
22	55.52	3,062	55.15	0.10	0.04	0.14	0.25
23	42.32	2,600	61.44	0.09	0.04	0.13	0.26
24	39.26	2,457	62.58	0.09	0.04	0.11	0.26
25	33.74	2,014	59.69	0.08	0.04	0.11	0.26
26	43.46	2,428	55.87	0.10	0.05	0.14	0.27
27	38.53	2,543	66.00	0.09	0.04	0.12	0.25
28	47.26	2,283	48.31	0.15	0.06	0.20	0.24
29	38.39	2,194	57.15	0.08	0.04	0.11	0.25
30	42.21	2,439	57.78	0.11	0.06	0.15	0.26
Mean	56.16	3,116	55.99	0.10	0.05	0.14	0.26

SNP, single-nucleotide polymorphism; MAF, minor allele frequency; SD, standard deviation.

### Estimation of LD

Table 1 summarizes the mean, median and standard deviation of *r*^2^ values for each coho salmon chromosome. All of the 93,502 SNPs placed onto chromosomes and which passed quality control were included in this analysis. These markers encompassed 1,684.62 Mb of the genome, representing ~71.1% of the total genome size (assuming a genome size of 2,369 Mb based on the total sequence length of the final assembly GCF_002021735.1). The molecular marker density per chromosome per Mb, ranged from 48.31 to 66 with a mean of 55.99. In general, SNPs were uniformly distributed along the 30 chromosomes. The number of SNPs on each chromosome ranged from 1,954 on Okis16 to 4,846 on Okis04, which is in agreement with Okis16 and Okis04 being the shortest and longest chromosome, respectively. The overall mean linkage disequilibrium (measured as *r*^2^) among SNP pairs was 0.10 ± 0.14. The global median was lower at 0.05. Low average LD among adjacent SNPs along the 30 chromosomes was observed in the current population, with values ranging from 0.07 to 0.15 (Table 1).

**Figure 1:**
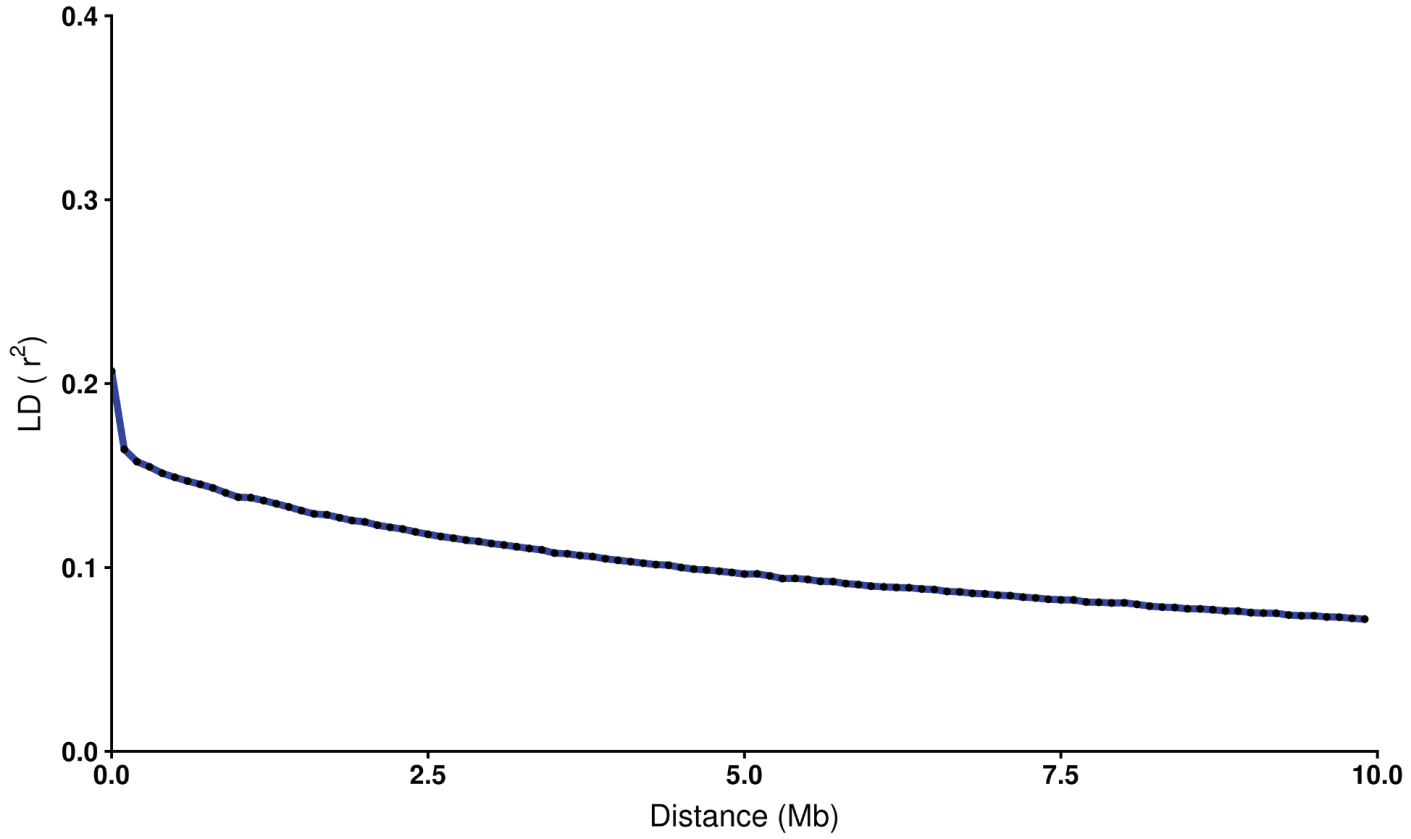
Decay of average LD (*r*^2^) over distance among SNPs in coho salmon (*Oncorhynchus kisutch*) population. The blue line shows the mean LD in each 100 kb sliding window. Each black dot represents average *r*^2^ within each bin.

To estimate the decay of linkage disequilibrium as a function of physical distance, SNP pairs were sorted into bins of 100 kb, and mean values of *r*^2^ were calculated for each bin. As observed in other species (Lu et al., 2012; Kijas et al., 2017; Vos et al., 2017), LD declines smoothly as the physical distance increases between markers (Figure 1). A maximum average LD of 0.21 was estimated for SNPs less than 1 kb apart. This value declines quickly at marker distances up to 0.1 Mb, with a value of 0.16. From 1 Mb to 10 Mb LD range from 0.14 to 0.07. The latter value represents the lowest average LD estimated in the current data set. The *r*^2^ estimation drops below 0.2 at a distance of ~40 kb.

**Figure 2:**
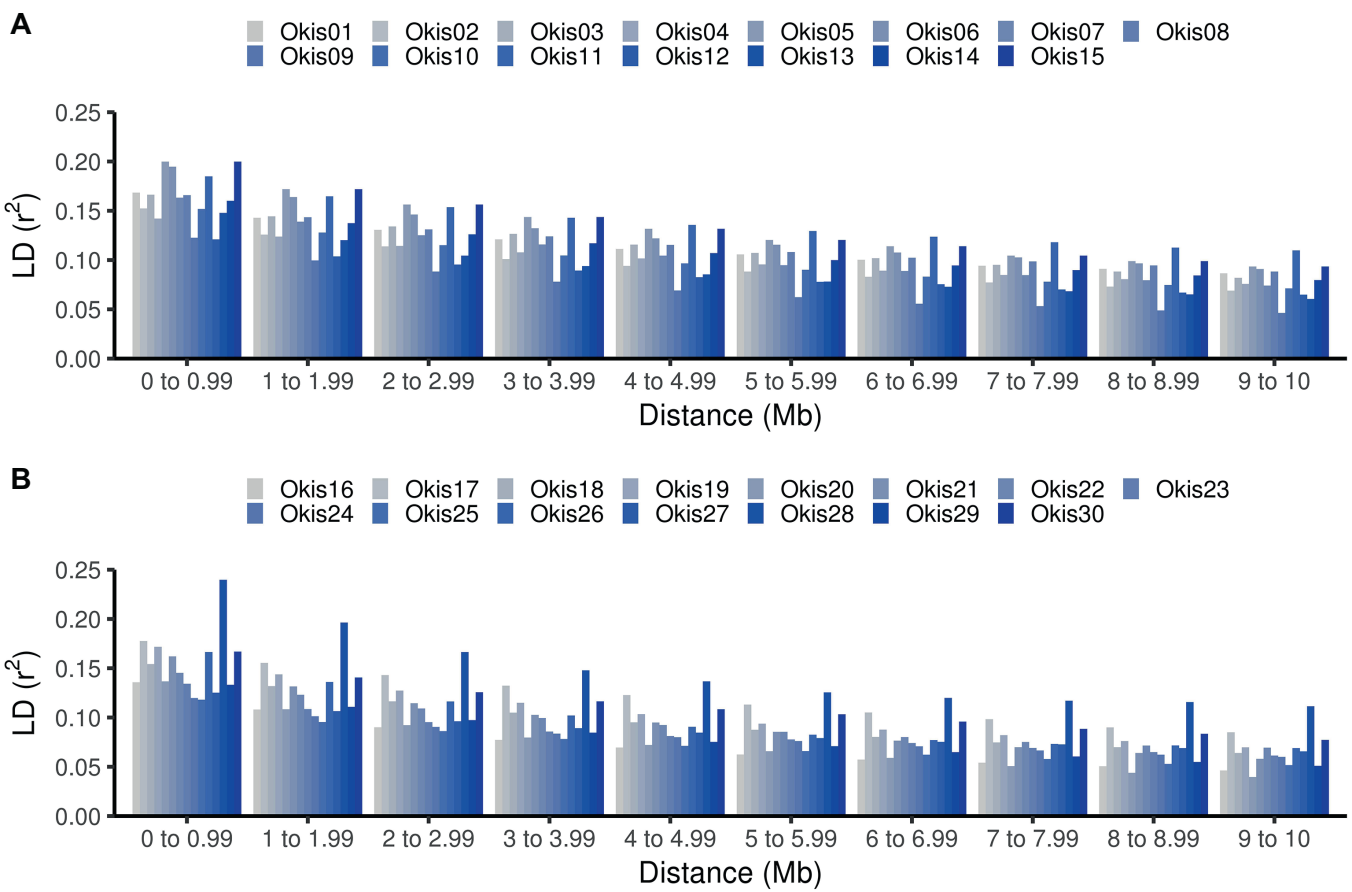
Linkage disequilibrium estimations along the 30 chromosomes of coho salmon. Average values of LD measured as *r*^2^ per chromosome, according to distances between SNPs. Estimated values are shown from Okis01 to Okis015 **(A)** and from Okis16 to Okis30 **(B)**.

Comparison of average LD at different distance bins for each chromosome shows higher variation at closer distance bins (Figure 2). Suggesting that estimations of genome-wide linkage disequilibrium based on few chromosomes may be biased (Khatkar et al. 2008). Lower estimates of LD (<0.13) were found in Okis09, Okis12, Okis24, and Okis25, while higher levels of LD (>0.20) were estimated for Okis05, Okis15, and Okis28. When the distance among SNPs increased over 4 Mb, average LD values drops below 0.15 for all chromosomes. Furthermore, average *r*^2^ values <0.10 were estimated for all chromosomes, except for Okis11 and Okis28 at distances greater than 7 Mb.

### Effective Population Size and Number of Chromosome Segments

**Figure 3:**
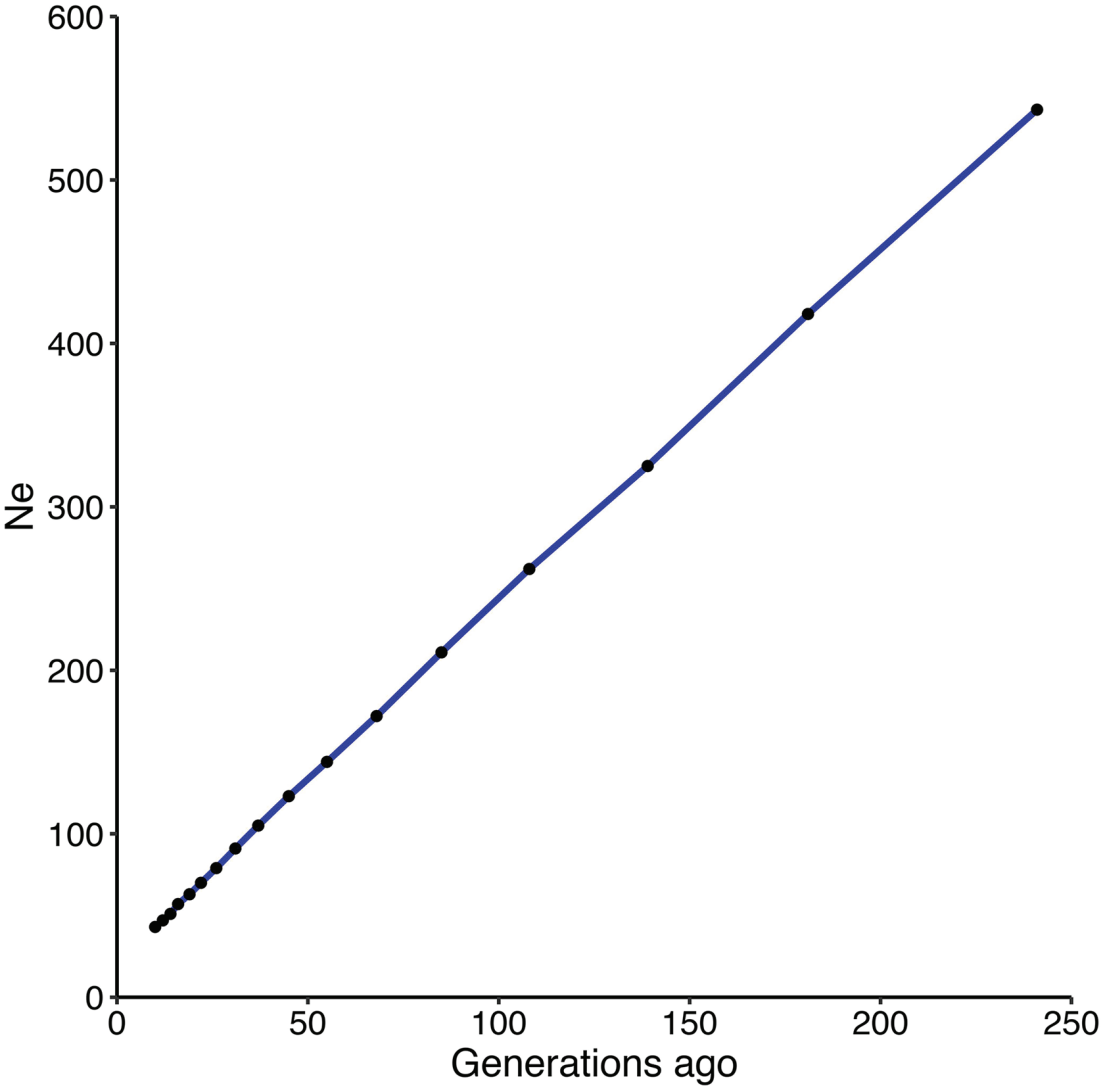
Effective population size estimation in coho salmon population. Estimates of effective population size (*N*_e_) over the past 241 generations based on the LD of an aquaculture strain of coho salmon. Black dots represent each *N*_e_ measurement.

Based on LD approach, estimated contemporary *N*_e_ reached up to 83.9. Figure 3 illustrates the estimated historical effective population size of the coho salmon, based on LD, from 10 to 241 generations ago. An increasing *N*_e_ as a function of the number of generation was observed, with a *N*_e_ of 43 estimated at 10 generations ago, and 543 for 241 generations ago.

Considering the estimated linkage disequilibrium (measured as *r*^2^) among all SNP pairs within each chromosome, we estimated that at least 74K SNPs markers would be needed for an efficient GWAs or GS analysis in the current farmed coho salmon population.

## Discussion

Understanding LD patterns enhances our knowledge of the demographic processes within the population. Biological factors such as recombination and mutation in conjunction with genetic drift, admixture and effective population size are important variables determining patterns of LD. For this reason, variation in LD among populations and genomic regions are of high interest and widely reported.

To our knowledge, this is the first study characterizing the whole-genome LD in a coho salmon population using a high-density SNP panel. The samples originated from the broodstock of a breeding program aimed at improving economically important traits for Chilean coho salmon aquaculture. Unrelated animals were chosen in order to avoid LD inflation that can occur when high kinship relationships are present in the sampled individuals (Gutierrez et al., 2015). Due to the increased bias of LD estimations, when estimating |D′| from small sample sizes (Bohmanova et al., 2010), we preferred to use the robust *r*^2^ statistic. Moreover, to predict the power of association mapping, *r*^2^ statistics is more useful. The minimum number of individuals necessary for an accurate *r*^2^ estimation has been suggested to range from 55 to 75 in cattle (Khatkar et al., 2008; Bohmanova et al., 2010). This range increases to 400 or more in case of |D′| (Khatkar et al., 2008). The number of individuals necessary to estimate LD depends on the demographic and genetic population history. Our sample size was within the range suggested above.

Sample sizes above 50 also provide accurate estimations of MAFs (>0.05) within a population, at a physical distance up to 10 Mb (Khatkar et al., 2008). Filtered markers showed an average MAF of 0.26 (ranging from 0.22 to 0.27) per chromosome (Table 1). A similar mean value was reported in Nellore cattle, ranging from 0.20 to 0.25 (Matukumalli et al., 2009; Espigolan et al., 2013) and from 0.28 to 0.30 in North American Holstein (Bohmanova et al., 2010). Despite that LD measurements of *r*^2^, tend to be less sensitive than |D′| to low MAF (Khatkar et al., 2008; Bohmanova et al., 2010; Kijas et al., 2017), low MAFs tends to underestimate LD measures (Espigolan et al., 2013). Thus, the high allele frequencies found in the current study suggest an unbiased estimation of our LD values.

Estimations of the extent and decay of linkage disequilibrium in the coho salmon breeding population provide insights into LD patterns in the coho salmon genome, which may have implications for GWAs, GS and for the design of SNP arrays. In terms of genomic predictions, the accuracy is influenced by multiple factors such as genetic architecture of the trait of interest, average relatedness between training and testing individuals, density of the SNP panel, effective population size, and effective number of chromosomes segments (*M*_e_) (Daetwyler et al., 2010; Pszczola et al., 2012). Due to LD, loci do not segregate independently within a finite population. Thus, *M*_e_ can be considered as the number of chromosome segment which segregates independently within a population. When a lower number of segments are estimated, independent parameters are needed to be estimated from the same dataset, i.e., higher accuracy of genomic predictions (Yvonne et al., 2016; Lee et al., 2017). Based on the relatedness among pair of animals, Goddard (2009) suggested that the effective number of chromosomes is 2*N*_e_L/Log(4*N*_e_L). However, this approach may produce an underestimated *M*_e_, leading to an overestimation on the EBVs accuracy (Goddard, 2009). Thus, we decided to used 1/mean(*r*^2^) (Goddard et al., 2011) which is a much more direct approach. Even that the 74K SNPs predicted for this coho salmon population, is much higher than the estimated in a farmed rainbow trout population, in which authors suggested at least 20K markers for an efficient GWAs and GS study (Vallejo et al., 2018), the SNP array used in the current study allows coverage of the whole genome. However, further studies are needed to elucidate the minimum number of markers necessary to achieve a higher accuracy predictions compared to pedigree-based models, in the current coho salmon breeding population.

The variation in the average and standard deviation in the LD among chromosomes found in the current study (Table 1), is partly explained due to variation in recombination rates along different chromosomes (e.g., local hotspots for recombination), decreasing as a function of an increase in chromosome length (Arias et al., 2009; Espigolan et al., 2013), which in turn generates variation in LD along the different chromosomes, as observed in Figure 2. Therefore, inferences based on single or only on few chromosomes might be biased and inferences regarding LD would be best when using genome-wide data. LD information from the population may allow researchers to reduce the number of required SNPs for a genomic analysis by excluding redundant SNPs (Khatkar et al., 2008). This can be done by identifying tag SNPs, using information from haplotype block structure, as was previously done in Holstein-Friesian cattle (Khatkar et al., 2007).

Average *r*^2^ values estimated in our study were higher than those estimated in a wild Finnish Atlantic salmon population, with values ranging from 0.015 to 0.037 (Kijas et al., 2017). However, farmed Tasmanian Atlantic salmon showed mean LD (measured as *r*^2^) values up to 0.67 for SNPs closer than 1 kb (Kijas et al., 2017), almost three times higher than in the current work (0.21). However, our estimation is similar as the one found in Chilean Atlantic salmon populations with European origin (Barría et al., 2018b). Some authors have found low linkage disequilibrium estimations in others Atlantic salmon populations, although these estimations were reported in units of recombination (Gutierrez et al., 2015) and using sliding windows of 20 SNPs (Johnston et al., 2014). The different estimation metrics make it difficult to compare directly with the current work. The origin of the current breeding coho population most likely involves two isolated wild populations (The Kitimat River and Oregon). The admixture of both founders populations could explain the induced long-range and reduced short-range LD (Pfaff et al., 2001) estimated in this Coho salmon breeding population. A similar pattern has also been suggested in a highly admixed Norwegian Atlantic salmon population (Ødegård et al., 2014) and in a recently admixed farmed rainbow trout population (Vallejo et al., 2018).

We found that historical effective population size shows a decline from 543 individuals, 241 generations ago, to 43 individuals 10 generations ago. Similar *N*_e_ pattern reduction has been observed in cattle populations (Villa-Angulo et al., 2009; Makina et al., 2015). Even though this is the first study aimed to estimate the effective population size of a coho salmon breeding population, caution must be taken when evaluating the estimations for the number of generations (Corbin et al., 2012). For recent generations, large c values are involved and do not necessarily fit the theoretical implications proposed by Hayes (Hayes et al., 2003) for *N*_e_ estimations. In the oldest generation, after 4*N*_e_ generations ago, none of the SNPs can be reliably sampled (Corbin et al., 2012). Therefore, *N*_e_ estimations after 4*N*_e_ generations ago may be questionable.

Contemporary effective population size calculated based on the LD is 83.9. This estimation is higher than the estimated for 10 generations ago (approximately 20 year ago, assuming an interval generation of 2 years), which agree exactly with the beginning of the breeding program of this farmed population (Neira et al., 2014). This value, is still below the minimum *N*_e_ value (500) suggested to retain the evolutionary potential and sustainability of a breeding program (Franklin and Frankham, 1998; Ponzoni et al., 2010). Thus, a modified mate allocation strategy that allows to increase the effective population size is suggested for the current breeding population.

## Conclusions

In the current study, we performed an LD analysis with 64 coho salmon genotyped with 93,502 SNPs. We showed the feasibility to estimate LD and infer the effective population size based on the observed LD using data from a high-density SNP array. Furthermore, based on the current effective population size, a new mate allocation strategy that allows to increase it is suggested.

## Ethics Statement

Coho salmon individuals and sampling procedures were approved by the Comité de Bioética Animal from the Facultad de Ciencias Veterinarias y Pecuarias, Universidad de Chile (Certificate N08-2015).

## Author Contributions

AB performed DNA extraction, LD and Ne analysis, and wrote the initial version of the manuscript. KC contributed on the data analysis and discussion. GY contributed with LD analysis and discussion. AJ performed DNA extraction. JPL contributed with study design. BK, JSL, and ER developed the chip array. WD contributed with writing and discussion. JMY conceived and designed the study, supervised work of AB, and contributed to the analysis, discussion, and writing. All authors have reviewed and approved the manuscript.

### Conflict of Interest Statement

The authors declare that the research was conducted in the absence of any commercial or financial relationships that could be construed as a potential conflict of interest.

## Footnotes

^1^http://www.sfu.ca/epic4/index.html
